# Physical Properties of Shellac Material Used for Hot Melt Extrusion with Potential Application in the Pharmaceutical Industry

**DOI:** 10.3390/polym13213723

**Published:** 2021-10-28

**Authors:** Guangming Yan, Zhi Cao, Declan Devine, Manfred Penning, Noel M. Gately

**Affiliations:** 1 Materials Research Institute, Technological University of the Shannon, Midlands Midwest, N37 HD68 Co. Westmeath, Ireland; g.yan@research.ait.ie (G.Y.); zhi.cao@tus.ie (Z.C.); declan.devine@tus.ie (D.D.); 2 Shellac Consultant, Wormser Strasse 28, D-55277 Oppenheim, Germany; manfred.penning@t-online.de

**Keywords:** shellac, hot melt extrusion, rheology, solubility enhancement, processability, DSC

## Abstract

Hot melt extrusion offers an efficient way of increasing the solubility of a poorly soluble drug. Shellac has potential as a pharmaceutical matrix polymer that can be used in this extrusion process, with further advantages for use in enteric drug delivery systems. The rheological property of a material affects the extrusion process conditions. However, the literature does not refer to any published work that investigates the processability of various shellac materials. This work explores various types of shellac and explores their physicochemical and thermal properties along with their processability in the hot melt extrusion application. Physicochemical characterization of the materials was achieved using differential scanning calorimetry, Fourier transform infrared spectroscopy, scanning electron microscopy and energy-dispersive X-ray spectroscopy. Additional processability characterization was achieved using melt flow index and rheology analysis. The results indicated that there was no chemical difference between the various shellac types compared in this study. However, the extrudable temperature ranges and rheological properties of different shellac types varied; SSB 55 Pharma FL had the lowest processing temperature and glass transition temperatures. Due to the shear-thinning behaviours, shellac can be extruded at lower temperatures. This study provides necessary data to determine the processing conditions in hot melt extrusion applications for the range of shellac materials.

## 1. Introduction

Shellac is the refined form of the natural resin LAC produced by the female insect Kerria Lacca. India, Thailand and southwest China are the main production areas of LAC [1,2]. Shellac is a complex mixture consisting of aliphatic and alicyclic acid components. Different types of insect species and host trees determine the composition of shellac material. However, there are no chemical differences between various shellac material, only the contents of each component are different [3]. In various pharmacopoeias, shellac is classified by the refining process as well as the chemical properties and acid values. Besides the acid value, the shellac quality can be characterised by its glass transition temperature, pK_a_ value and intrinsic dissolution rate [4,5]. The use of shellac material can be traced back to ancient times; the best-known applications were as the binding material in music records and also used to prepare varnishes as the protective layer on artistic objects. However, it was replaced by synthetic polymer due to its brittleness [6,7,8,9,10,11]. Various types of shellac were developed to expand its application field by the addition of some additives or surfactants [10].

Because shellac is a non-toxic and physiologically harmless material, the US Food and Drug Administration (FDA) has listed shellac as Generally Recognized as Safe (GRAS) material. With this fact, as well as its excellent film-forming and moisture-resistant properties, shellac has been widely used in the medicine and food industries as a coating material [12,13,14,15]. In the pharmaceutical industry, shellac was employed as an enteric coating material due to its pH-sensitive properties [16,17,18,19]. Pearnchob stated that shellac could provide the same water resistance and taste masking with a lower coating level [20]. Moreover, Ansari et al. (2013) made blends of shellac with novolac, which resulted in improved film properties. The new film showed improvement in gloss and impact resistance [21]. Silva et al. (2016) found that the encapsulated probiotic under simulated gastrointestinal conditions can be effectively protected by an alginate-shellac blend combined with coconut fat [22]. Additionally, Pearnchob et al. (2003) and Sontaya et al. (2008) investigated the feasibility of extended-release shellac-matrix tablets prepared by either compression of powder or wet granule methods [20,23]. In 2017, Gately et al. investigated targeted delivery of probiotics prepared by melt extrusion technology [24].

In modern industry, hot melt extrusion is a well-known, efficient industry processing technology. It forces raw materials through a die under controlled conditions to produce a product of uniform shape and density [25,26]. Over the last few years, hot melt extrusion has been inducted into the pharmaceutical industry [27]. It has long been known that twin-screw extrusion can increase the solubility of drugs by the formation of a solid dispersion [25,28]. Since Sekiguchi and Obi (1961) created the first solid dispersion, it has become the most successful method for enhancing the bioavailability of BCS class II compounds [29,30].

In the extrusion process, the material undergoes various temperatures and shear rates. Moreover, the melt behaviour of material in the extrusion process is significantly affected by the viscosity of the molten polymer. The processability of the material is appreciably affected by the way the polymer reacts with flow and deformation [31]. When determining the extrusion parameters before processing, for the polymers with high viscosity, they always require higher melt temperature profiles and higher shear rates in the barrel. Furthermore, discharge pressure and more power consumption are often required. This would help to reduce the number of trials needed to find the desired conditions for extrusion [32,33,34,35].

Following a review of the literature, it is clear that shellac is widely used in pharmaceutical and food industries as a coating material. However, the authors did not find a reference to any published work that investigates the processability of shellac material. As a result, this work will explore four different types of shellac (Dewaxed Shellac AFS HS 700K, AFS Shellac WL, AFS Shellac RTH and SSB 55 Pharma FL). From the material data sheet, all shellac materials used in this study were sourced from the same seedlac Kushmi type. However, refining processes are different, which results in different applications. Dewaxed shellac HS 700K and SSB 55 Pharma Fl are pharmaceutical grade and have a lower color index number because of the physical bleaching process. AFS shellac WL and AFS shellac RTH contain wax; they are refined from the same seedlac as the other two shellac types, but the hot melting process is used on a different date, which does not remove the naturally occurring wax contained in the Lac. In this study, the authors investigate the physicochemical properties and processability with the subsequent view of using shellac as a matrix polymer for enteric-targeted drug delivery systems [36,37].

## 2. Materials and Methods

### 2.1. Material

The shellac materials used in this study are shown in Table 1. Dewaxed Shellac AFS HS 700K, AFS Shellac WL and AFS Shellac RTH were received as gifts from A.F. Suter (Witham, UK). Shellac SSB 55 Pharma FL was received as a gift from SSB (Stroever Schellack, Bremen, Germany). All other reagents and solvents used were of analytical grade.

### 2.2. Grinding

Ground shellac was prepared by milling shellac flakes in a Planetary Mono Mill PULVERISETTE 6 (FRITSCH, Idar-Oberstein, Germany) classic line and sieving through a 500 μm mesh sieve. During the grinding process, a short grinding operation time of 30 s was employed to prevent the melting during milling. Ground shellac was used for the melt flow rate test; determination of thermal properties including glass transition temperatures (Tg), melt temperature and decomposition temperature (Td); Fourier transform Infrared spectroscopy (FT-IR) and rheology properties.

### 2.3. Differential Scanning Calorimetry (DSC)

A TA instrument DSC 2920 DSC (TA Instruments, New Castle, DE, USA) was used throughout the work. Samples of between 4 and 6 mg were weighed out using a Sartorius scale having a resolution of 1 × 10^−5^ g. Samples were then placed in non-perforated aluminium pans, which were crimped before testing, with an empty crimped aluminium pan being used as the reference cell. Volatiles were removed from the purging head with nitrogen at a rate of 30 mL/min. Calibration of the instrument was performed using indium as standard. Temperature ramp was from −30 to 120 °C at 10 °C/min. An isothermal step of 1 min at 120 °C was introduced to remove excessive water. The T_g_ and T_m_ were determined from the second heating run by TA Universal Analysis 2000 software version 4.5A (TA Instruments, New Castle, DE, USA) based on Standard ISO 11357-2 2020 [38]. The half-step-height method was used to determine the glass transition temperature.

### 2.4. Attenuated Total Reflectance Fourier Transform Infrared (ATR-FTIR) Spectroscopy

Attenuated total reflectance Fourier transform infrared spectroscopy (ATR-FTIR) Indexer (PerkinElmer, Waltham, MA, USA) was carried out on a Perkin Elmer Spectrum One fitted with a universal ATR sampling accessory. All data were recorded at room temperature, in the spectral range of 4000–650 cm^−1^, utilizing a 16 scan per sample cycle and a fixed universal compression force of 70N. Subsequent analysis was carried out using OMNIC Spectrum software version 9.2.86 (Waltham, MA, USA).

### 2.5. Melt Flow Index (MFI)

Melt flow index values of the four different types of shellac were assessed using a fixed weight of 1.2 kg with a CEAST Melt Flow Quick Indexer (Instron Norwood, MA, USA) under ASTM D1238-10. The melted material flowed through an orifice of 2.095 mm diameter for 10 min, and the values were reported in g/10 min. All samples were tested at 90 °C.

### 2.6. Rheometer

Melt rheological experiments were carried out in an oscillatory mode, on a rotational rheometer TA Discovery HR-2 hybrid Rheometer (TA Instruments, New Castle, DE, USA) equipped with a parallel plate (25 mm diameter). All samples were measured at various temperatures (70 °C, 80 °C, 90 °C) with 500 μm gap distance. Four types of experiments were carried out in this study. For the first experiment, oscillatory time sweeps were characterized at 90 °C and with constant angular frequency of 1 Hz and 1% strain. The second experiment was amplitude sweep performed with a strain range of 0.01% to 100% at an angular frequency of 1 Hz. The third experiment used oscillation frequency analysis with a constant strain of 1% and angular frequency performed with a range of 0.1 to 100 Hz. The final experiment was oscillation temperature sweep, where samples were heated from 65 to 110 °C with a heating ramp rate of 3 °C/min at a frequency of 1 Hz with a strain value of 1%. The instrument was calibrated before each test.

### 2.7. Dissolution Test

Dissolution testing was carried out using a Sotax AT7 smart dissolution system from Carl Stuart Ltd. (Sotax Corporation, Aesch, Switzerland). Tests were carried out using the Paddle method (USP XXV). The dissolution media used in these tests consisted of buffer solutions (pH 1.2, pH 7.4). All tests were carried out at 37 ± 0.5 °C. The stir rate was set to 100 rpm with 900 mL of dissolution media used per vessel. The wavelength and absorption of 100% shellac concentration were determined using a SHIMADZU UV-1280 UV–Vis Indexer (Shimadzu, Kyoto, Japan) spectrophotometer. In the case of melt flow index extruded samples, test specimens of constant size and surface area were produced by cutting the extrudate strands manually to give granules of length 1 cm and accurately weighed using a Sartorius scale having a resolution of 1 × 10^−5^ g. Samples were manually taken every 30 min, filtered with the glass microfiber filters (1.2 μm), recorded spectrophotometrically at 221 nm using a SHIMADZU UV-1280 UV spectrophotometer (Kyoto, Japan), and all the tested samples were returned to the dissolution vessel after reading. The dissolution profile was observed from a plot of time versus concentration in percentage.

## 3. Results and Discussion

### 3.1. IR Spectrum

FTIR spectroscopy was employed to investigate what chemical bonds were present in each material. Infrared spectroscopy is useful because the peak position in an infrared spectrum correlates with the molecular structure [39]. In this study, FTIR was used to compare each of the four shellac materials and search for visible differences in molecular structure.

**Figure 1:**
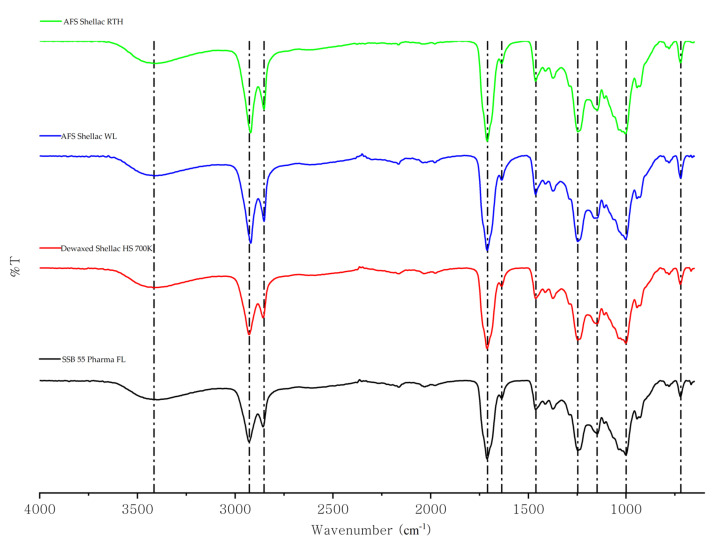
FTIR spectrum of different types of shellac.

Figure 1 shows the FTIR spectra of four different kinds of shellac. It can be seen that all the existing peaks of various shellac were almost identical and corresponded to those reported in the literature. There was a clear broad peak in the range of 3700–3200 cm^−1^ with a maximum at 3416 cm^−1^, which was attributed to the –OH vibrations from acidic and hydroxylic functional groups [34,40], as well as a strong absorption band at 2928–2920 cm^−1^ and 2852 cm^−1^, which represented –CH stretching [41]. The carbonyl band from the acid formation was visible at 1710 cm^−1^ with a slight shoulder at 1636 cm^−1^, corresponding to the C=O band of an ester [13,42]. This region on the right-hand side of the diagram (from about 1500 to 600 cm^−1^) is called the fingerprint region. It can be used to identify unknown or two different organic compounds by comparing their fingerprint spectroscopy graphs [43]. In Figure 1, the main absorption bands in the fingerprint region were at 1462 cm^−1^ (CH_2_ bend), 1374 cm^−1^ (CH_3_ bend), 1246 cm^−1^ ((C–O) stretch from ester), 1148 cm^−1^ (C–O stretch from acid), a broadband between 1010 and 1000 cm^−1^ (C–O stretch from alcohol), 943 cm^−1^ (C–H stretch/CH_2_ from alkenes) and a weak peak visible at 720 cm^−1^, which are the characteristic peaks of wax, representing CH_2_ rocking from shellac wax [44,45]. Table 2 shows the absorbance of each shellac material at 720 cm^−1^. The intensity difference of the samples is simply because AFS Shellac RTH and AFS Shellac WL have higher wax concentrations, which are around 4–5%. It is clear that even though SSB 55 Pharma FL and Dewaxed Shellac HS 700K are dewaxed shellac, there was still some traces of wax detectable in the sample.

The IR result showed that the received shellac is a mixture containing ester, acid and hydroxyls groups, which refer to the resin part of the composition [46]. Moreover, all the similar characteristic peaks observed showed there is no chemical difference between the four shellac types as all are Kushmi based. The main difference between each shellac sample was the content of each ingredient. As a result, it is not conceivable to identify each sample using its FTIR spectrum [24].

### 3.2. Melt Flow Index Analysis

Melt flow index (MFI) is a conventional and easy method used to measure the flow rate of a polymeric material through an orifice of specified length and diameter under the prescribed conditions of temperature and pressure [47,48]. MFI can determine the quality of a polymer, relate its flow properties to its application and measure the viscosity of the polymer in a molten state [49,50]. In modern industrial process technology, the shear stresses used during the production cycle are much higher than the applied shear stresses or resultant shear rates in this bench test, indicating that the data obtained from MFI analysis do not necessarily correlate with the processability of the polymer [51]. However, it does provide useful information about how each material flows when they are processed. Generally, a low molecular weight material is more amenable to flow than a high molecular weight material [34,52]. Thus, higher MFI values indicate the material flows better at the tested temperature. Generally, if one material has a high MFI value, it may most often be chosen as the raw material when the industry process involves high rates of shear, such as in HME [47].

In this study, each type of shellac had a different MFI value. Shellac SSB 55 Pharma FL had the highest figure, 11.88 g/10 min, compared to other shellac batches. AFS Shellac RTH also had a high MFI value, 9.45 g/10 min. Nevertheless, AFS Shellac WL had the lowest MFI value (3.00 g/10 min), which means that compared to other shellac materials, AFS Shellac WL had the most resistance to flow in the test conditions. From the IR result, little to no chemical difference between various shellacs was observed; the only difference was the content of each ingredient was different owing to their origin from different types of seedlac and their refining processes [4]. According to the material certificate of analysis documents, AFS Shellac RTH and WL were refined by the same refining process and same raw materials, but with different processing parameters and different manufacturing dates, which resulted in their different flow viscosities and different grades of ageing. Moreover, Dewaxed Shellac HS 700K and SSB 55 Pharma FL were purified from the same type of seedlac (Kushmi seedlac) but by different company refining processes. Dewaxed shellac HS 700K was produced earlier, where the MFI value of dewaxed Shellac HS 700K was approximately half of the SSB 55 MFI value. This shows the ageing process will significantly affect the properties of shellac. Moreover, according to the material certificate of analysis documents, AFS Shellac WL had the highest wax content. The presence of wax will restrict the flow of the material, which resulted in the lower MFI value [53].

### 3.3. Rheology

Hot melt extrusion (HME) as a continuous manufacturing process is widely used in the plastic and rubber industries to manufacture a broad variety of products [54,55]. Compared to other methods, it has many benefits, such as no solvents are required during the process, and it may involve less process step. Recently, the pharmaceutical industry has demonstrated great interest in the HME process [50,56]. In this study, shellac was processed by HME technology. Therefore, a good understanding of rheological properties is very important. It not only investigates flow and deformation properties of a material at the specific settings and conditions, but also insight into the underlying molecular structure of the polymers is gained by comparing their viscoelastic properties [57]. Moreover, it allows us to determine the conditions of shellac processing [58]. In this study, steady-state rheometry was performed on all four shellac samples. The rheological behaviour of a polymer material may be affected by many factors [59].

The first set of rheology tests performed was oscillatory time sweeps. This test directly provides the necessary information about how material changes as a function of time. Before any subsequent rheological testing, it is important to identify that the material properties do not change during the test period at the constant test temperature of 90 °C. The result of oscillatory time sweep on shellac material shows that the material’s storage modulus (G’) remained at a steady value. This indicates that the material’s structure was not altered during the 900 s testing period at 90 °C.

When the polymer material is under a critical stain, its rheological properties remain at a steady-state value. That range is called linear viscoelastic region (LVR) [60,61]. When the applied strain is higher than the linear viscoelastic region, the material structure would be destroyed, and its response would be non-linear. In addition, the storage modulus would begin to decrease [62]. As a result, determining the linear viscoelastic region of an unknown material would be the second necessary step in rheology analysis [61].

**Figure 2:**
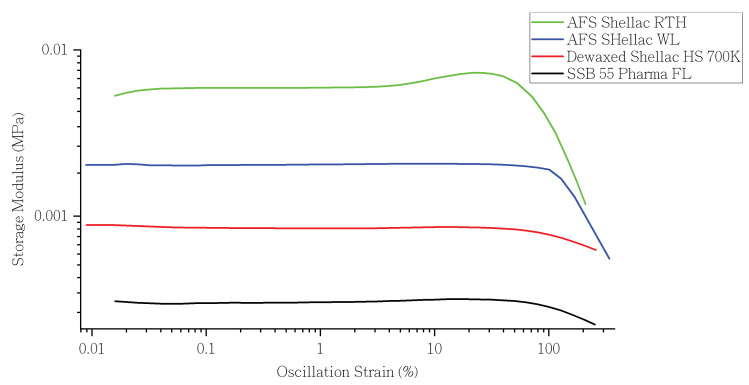
The storage modulus of various shellac materials versus oscillation strain (%) in amplitude sweep at 90 °C.

A strain sweep was used to determine the material’s linear viscoelastic region (LVR). Figure 2 overlays the results of amplitude sweep of each shellac material at 90 °C. Additionally, Table 3 illustrates the linear viscoelastic region (LVR) of shellac materials at different temperatures. The test procedure could not be completed when the analysis is running at 60 °C, or even lower temperature, as the material was too viscous to flow, which exceeded the test range of the machine. Except for AFS Shellac RTH, all other types of shellac had a narrow LVR at low temperatures.

At a lower temperature, AFS Shellac RTH had a higher LVR range compared to the other three types of shellac, which is likely due to the existence of wax in the AFS Shellac RTH, acting as plasticiser when it is not in a molten state [63]. However, when the temperature increased to 80 °C, shellac SSB 55 Pharma had a wider LVR range, nearly twice that of Dewaxed Shellac AFS HS 700K and AFS Shellac WL.

**Figure 3:**
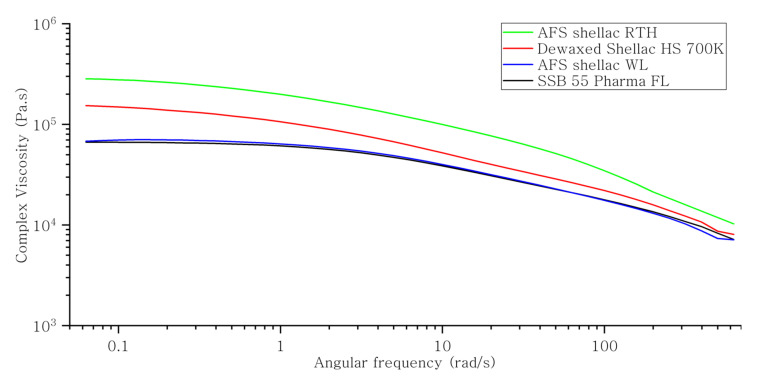
Complex viscosity versus frequency of various shellac in frequency sweep at 70 °C.

**Figure 4:**
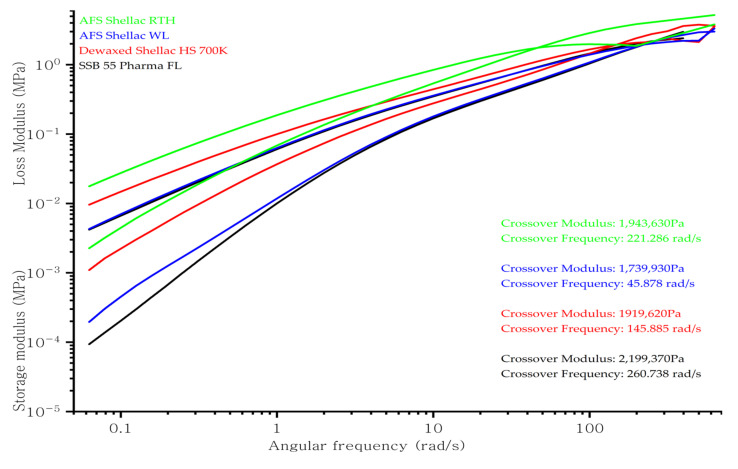
The storage modulus and loss modulus versus frequency of various shellac in frequency sweep at 70 °C.

During melt extrusion, the materials undergo vigorous mixing under pressure and shear rate, which accelerates dissolution of one component into the other in a drug/polymer mixed system. Therefore, it is necessary to ascertain the effect angular frequency would have on the viscosity of mixtures at a defined temperature [64]. As a result, oscillation frequency analysis is required to find out the effects of angular frequency on complex viscosities for each shellac at 70 °C. As shown in Figure 3, the viscosity was first determined at the given temperature at the angular frequency of 0.1 Hz, which was then increased gradually up to 100 Hz. The viscosity of shellac material in this study was found to follow a shear-thinning behaviour typical of many polymer responses, as shown in Figure 4. In all cases, with the increase in shear rate (indicative of processing speed) the viscosity of shellac decreased [34]. Moreover, AFS Shellac WL alone had the highest melt viscosity of all shellac types tested, which indicates the existence of wax, directly affecting the viscosity of the shellac at lower temperature. This result correlates well with results obtained using MFI analysis. Furthermore, in all cases, there was a much sharper decrease in viscosity with the increase in angular frequency. The profound drop in viscosity for AFS Shellac WL may attributed to the larger amount of wax initially present in the shellac. The viscosity reduction in Dewaxed Shellac HS 700K is much higher than Shellac SSB 55 Pharma FL, which may be because of the different processing parameters used by different supplier companies and the earlier manufacture date.

Figure 4 illustrates the storage and loss modulus of the shellac in frequency sweep analysis at 70 °C. Typically, the molecular weight (Mw) and the molecular weight distribution (MWD) of the material would affect the crossover point of the modulus in frequency sweep analysis. When the polymer has a higher molecular weight (Mw), the crossover point will move to lower frequency compared to a low Mw. Alternatively, when the polymer has narrow MWD, the crossover point would shift to higher modulus values [65]. The result indicates that AFS Shellac WL had a lower Mw compared to other shellac materials assessed. Moreover, shellac SSB 55 Pharma FL had the narrowest MWD [31].

**Figure 5:**
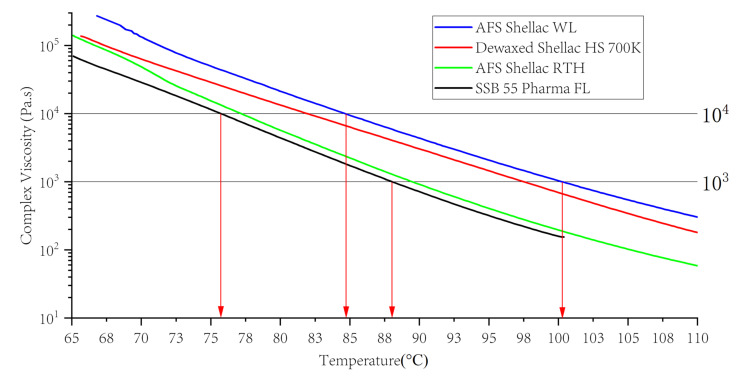
Viscosity versus temperature of various shellac.

When using hot melt extrusion technology to process the polymer, the materials undergo a programmed temperature profile. As a result, it is necessary to determine what effect temperature has on the viscosity of mixtures across a range. Temperature ramp analysis investigates how increasing thermal energy affects the material’s viscosity and also the materials melt strength [66,67]. The results of the temperature ramp of four types of shellac at a constant strain and frequency are shown in Figure 5. The viscosity was first determined at the given strain at the temperature of 65 °C, which was then increased gradually up to 110 °C with a heating rate of 3 °C min^−1^. Figure 5 illustrates that all the shellac materials were sloped almost identically as a function of temperature at an angular frequency of 1 Hz. With the temperature increasing the viscosity of shellac decreases. Moreover, the AFS Shellac WL alone had the highest melt viscosity when compared to all other shellacs, which suggests that the wax affects the viscosity of the shellac. Additionally, in all cases, there was a much sharper decrease in viscosity with an increase in temperature. The profound drop in viscosity for AFS Shellac WL may be attributed to the larger amount of wax initially present in the shellac, which melted gradually when the temperature increased. This experiment provided evidence that the viscosity of shellac SSB 55 pharma FL changed less as the temperature changed.

From an earlier report by other researchers, the extrudable viscosity range for a polymer material was reported to be 1000 to 10,000 Pa s [66]. This range is determined by one simple rule: the viscosity of the polymer should be low enough for the drug to dissolve in it and high enough for the extrusion process to occur. For AFS Shellac WL, Dewaxed Shellac AFS HS 700K, AFS Shellac RTH and shellac SSB 55 Pharma FL, the temperatures corresponding to the viscosity of 1000 to 10,000 Pa s were 85 to 100 °C, 82 to 97 °C, 77 to 89 °C and 75 to 87 °C, respectively; therefore, the extrudable temperature range for various types of shellac was different [68]. However, due to the existence of shear-thinning behaviours, each shellac material may be extruded at lower temperatures.

### 3.4. Differential Scanning Calorimetry (DSC)

**Figure 6:**
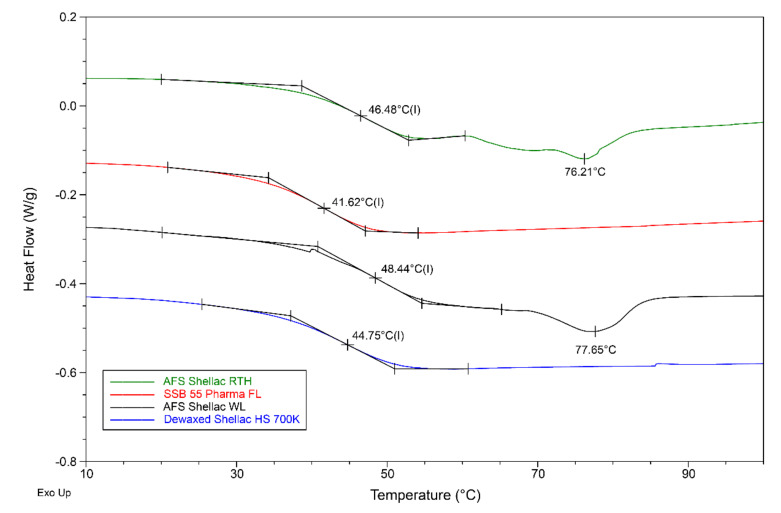
Overlaid DSC result of original shellac samples.

Glass transition temperature (T_g_) is one of the fundamental properties of any material, and it is essential in material processing and design, especially for an amorphous polymer [69]. The glass transition is also known as glass–liquid transition, and it is the reversible process in an amorphous material or semi crystalline material where the polymer moves from the solid state into a rubbery state with increasing temperature, meaning the polymer molecular structure begins to become flexible [70]. The heat capacity of the various shellac resins changed during the glass transition process, and DSC is a useful method to determine the glass transition temperature [71,72]. However, the transition does not occur at a specific temperature for a short time but somewhat over a temperature range, as shown in Figure 6. In this study, the half-step-height method was used to determine the T_g_ value. The shellac materials investigated in this study were proposed as potential matrix materials for drug delivery systems. As such, it is advantageous that the glass transition temperature of the selected material is well above the storage or drug release temperature [68].

Differential scanning calorimetry can record data of the overall heat flow as a function of temperature. The study of the thermal behaviour of shellac was carried out using DSC analysis. In matrix-assisted laser desorption ionization mass spectroscopy (MALDI-MS) measurements, shellac consists mostly of monomeric and oligomeric compounds, though the glass transition still can be observed [73]. Below its T_g_ value shellac is a hard, brittle, amorphous substance, and above its T_g_ value shellac becomes a soft and flowable thermoplastic [4]. As it can be seen in Table 4, as well as Figure 6, all T_g_ values were in a range between 41 °C and 49 °C. All shellac grades showed relatively similar thermal behaviour, had a single glass transition temperature, and the onset temperature of glass transition process was perceptible. SSB 55 Pharma FL, based on Kushmi seedlac, had the lowest T_g_ (41.62 °C). The T_g_ value of Shellac HS 700K, refined by solvent extraction and based on Kushmi seedlac but with an early production date, was at 44.75 °C, which is higher than SSB 55 Pharma FL. Moreover, because of the existence of wax, the two wax-containing shellac grades AFS Shellac RTH and AFS Shellac WL, both refined by a melting process based on Kushmi seedlac, had T_g_ values much higher than the dewaxed shellac, which were 46.48 °C and 48.44 °C, respectively. This result corresponds well to the MFI test and rheology analysis where AFS Shellac WL had the lowest MFI value, but the highest viscosity and T_g_ value [5]. Nonetheless, the two wax-containing shellac batches had a noticeable small melting peak around 76 °C to 78 °C. This melting peak was the result of the shellac wax melting when the sample was heated [74].

### 3.5. Dissolution

**Figure 7:**
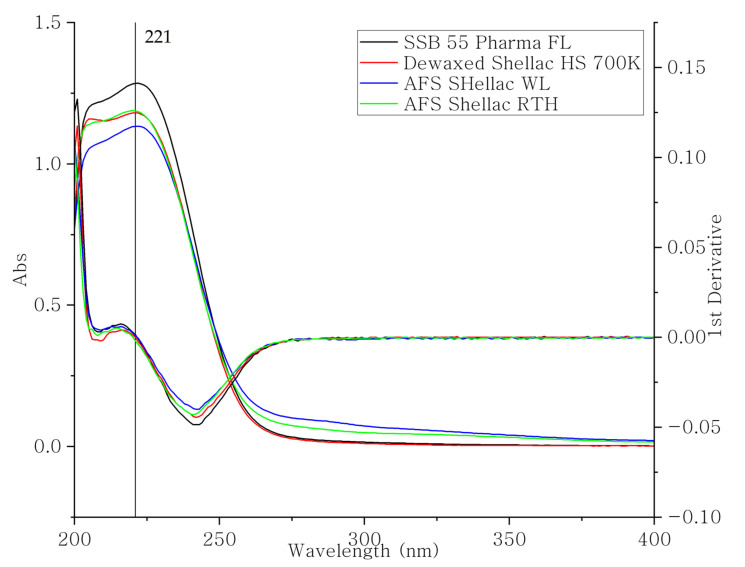
The UV spectrum and its 1st derivative curve of four different types of shellac.

In order to use a spectrophotometer to record the shellac dissolution profile, the wavelength and absorption of 100% shellac concentrations were determined first. As shown in Figure 7, all types of shellac showed similar spectrum curves, which is further confirmed with IR results. All types of shellac materials exhibited little chemical differences. The differences between shellac types were caused only by different amounts of their various constituent ingredients and not by their bulk structure. All of them had a maximum UV absorption at 221 nm independent of shellac type. The standard calibration line of each shellac types was determined depending on the investigated batch. In this study, the investigation was focused on the raw material without any drug loading; spectrophotometric detection was a suitable method for recording the dissolution profiles. From previous literature reviews, shellac is a weak acid. The dissolution profiles were expected to be pH dependent. Furthermore, because the nature of shellac is the same, the pH dependence was suitable for all samples. With increasing pH value, the dissolution rate and the amount of dissolved shellac increased.

**Figure 8:**
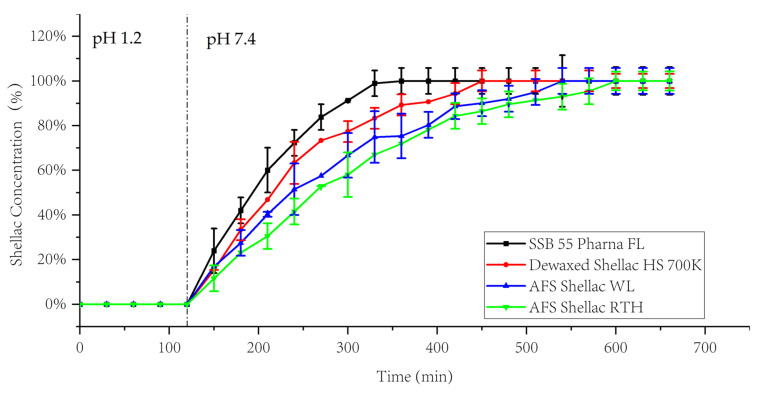
Dissolution profiles of the investigated shellac types at various pH.

The recorded dissolution profiles of shellac in different pH are shown in Figure 8. All types of shellac illustrated similar dissolution behaviours. At pH 1.2 no dissolution occurred. When the media changed to pH 7.4 PBS, the dissolution rate and the amount of dissolved shellac increased. From the result, shellac SSB 55 Pharma FL had the highest dissolution rate in any pH value compared to the others, which was expected, because of its lower T_g_ and acid value [5]. Moreover, as the dissolution process progressed, the dissolution rate decreased. Compared to the two wax-containing shellac types, dewaxed shellac had a higher dissolution rate, which is attributed to the wax being hydrophobic and cannot be dissolved in aqueous media. At pH 7.4, shellac SSB 55 Pharma Fl showed complete dissolution after 3.5 h, and Dewaxed Shellac HS 700K completely dissolved after 5.5 h. AFS Shellac RTH had the longest dissolution time, being completely dissolved after 9.5 h. However, at pH 7.4, all shellac materials dissolved completely.

### 3.6. Suggestion for Hot Melt Extrusion Process Conditions

From the DSC result, the glass transition temperature of the material was determined to be in the range of 41 °C and 49 °C. This means the temperature profile for the processing must be below 40 °C at the feeding zone and rise above the T_g_ of the material, along the extruder barrel to the die. Otherwise, the material would melt in the first conveying area, which is undesirable. Moreover, from the rheology analysis, all shellac materials exhibited shear thinning behaviour, which indicates that the processing temperature can be reduced at higher screw speed.

## 4. Conclusions

This work explores various types of shellac and investigates their physicochemical properties and processability as potential matrix materials for enteric-targeted drug delivery systems. Physical, thermal and rheology analyses of the shellac materials were conducted to support its application in hot melt extrusion as a potential drug delivery platform. All shellac materials were amorphous and had no difference in chemical structure by FTIR. During DSC analysis, the existence of a peak in two wax-containing shellac materials was due to the presence of the wax. The viscosity of the various shellac materials in this study was shown to follow shear-thinning behaviour, typical of many polymer responses, and correlated well with the melt flow index value. Moreover, based on the rheology results, the processing temperature ranges of different types of shellac for melt extrusion could be determined, and the extrudable temperature ranges were varied. Nevertheless, due to the shear-thinning behaviours, shellac can be extruded at lower temperature. Additionally, compared to the other shellac materials, shellac SSB 55 Pharma had the lowest process temperature, which indicates the highest processability. This study provides necessary data to determine the process conditions for the hot melt extrusion process involving shellac material. The content of wax and aging have significant effects on the physical properties of shellac. However, the aging behaviour of shellac is based on the self-esterification of one compound of the natural resin: aleuritic acid. This difference can be observed by gas chromatography–mass spectrometry technology. As a result, future studies are recommended to use GC-MS or HPLC/MS technology to identify the chemical content differences of the various types of shellac.

## Figures and Tables

**Table 1:** Shellac materials used in this study.

Batch Name	Type	Seedlac	Refining Process	Bleaching Process	Wax Containing	Manufacture Date
Dewaxed Shellac AFS HS 700K	Dewaxed shellac	Kushmi	Solvent extraction	Activated carbon	Less than 0.2%	16 February 2017
SSB 55 Pharma FL	Dewaxed shellac	Kushmi	Solvent extraction	Activated carbon	Less than 0.2%	August 2017
AFS Shellac WL	Wax containing shellac	Kushmi	Melting process	N/A	4.5%	05 June 2017
AFS Shellac RTH	Wax containing shellac	Kushmi	Melting process	N/A	3.6%	13 December 2017

**Table 2:** Shellac material used in this study.

Name	AFS Shellac RTH	AFS Shellac WL	Dewaxed Shellac HS 700K	SSB 55 Pharma FL
Absorbance at 720 cm^−1^	0.026	0.03	0.021	0.02

**Table 3:** The linear viscoelastic region of shellac at various temperatures.

	Temperature	60 °C	70 °C	80 °C	90 °C
Sample					
AFS Shellac WL		N/A	0.2 ± 0.01	30.8 ± 0.8	78.9 ± 0.4
Dewaxed Shellac AFS HS 700K		N/A	0.1 ± 0.01	33.7 ± 0.6	82.6 ± 1.0
Shellac SSB 55 Pharma FL		N/A	0.1 ± 0.004	64.2 ± 1.6	85.5 ± 1.0
AFS Shellac RTH		N/A	15.3 ± 0.3	51.0 ± 0.2	68.4 ± 0.8

**Table 4:** Glass-transition temperatures and melting temperatures of different shellac grades.

Material	T_g_ (°C)	T_m_ (°C)
Shellac SSB 55 Pharma F	41.7 ± 0.1	-
Dewaxed Shellac HS 700K	44.6 ± 0.1	-
AFS Shellac RTH	46.4 ± 0.1	76.5 ± 0.3
AFS shellac WL	48.2 ± 0.2	77.5 ± 0.2

## Data Availability

Not applicable.
